# Pectoral girdle and forelimb musculoskeletal function in the echidna (*Tachyglossus aculeatus*): insights into mammalian locomotor evolution

**DOI:** 10.1098/rsos.181400

**Published:** 2018-11-14

**Authors:** Sophie Regnault, Stephanie E. Pierce

**Affiliations:** Museum of Comparative Zoology and Department of Organismic and Evolutionary Biology, Harvard University, 26 Oxford Street, Cambridge, MA, USA

**Keywords:** biomechanics, model, glenohumeral, monotreme, range of motion, moment arm

## Abstract

Although evolutionary transformation of the pectoral girdle and forelimb appears to have had a profound impact on mammalian locomotor and ecological diversity, both the sequence of anatomical changes and the functional implications remain unclear. Monotremes can provide insight into an important stage of this evolutionary transformation, due to their phylogenetic position as the sister-group to therian mammals and their mosaic of plesiomorphic and derived features. Here we build a musculoskeletal computer model of the echidna pectoral girdle and forelimb to estimate joint ranges of motion (ROM) and muscle moment arms (MMA)—two fundamental descriptors of biomechanical function. We find that the echidna's skeletal morphology restricts scapulocoracoid mobility and glenohumeral flexion–extension compared with therians. Estimated shoulder ROMs and MMAs for muscles crossing the shoulder indicate that morphology of the echidna pectoral girdle and forelimb is optimized for humeral adduction and internal rotation, consistent with limited *in vivo* data. Further, more muscles act to produce humeral long-axis rotation in the echidna compared to therians, as a consequence of differences in muscle geometry. Our musculoskeletal model allows correlation of anatomy and function, and can guide hypotheses regarding function in extinct taxa and the morphological and locomotor transformation leading to therian mammals.

## 1. Background

**Figure 1.**
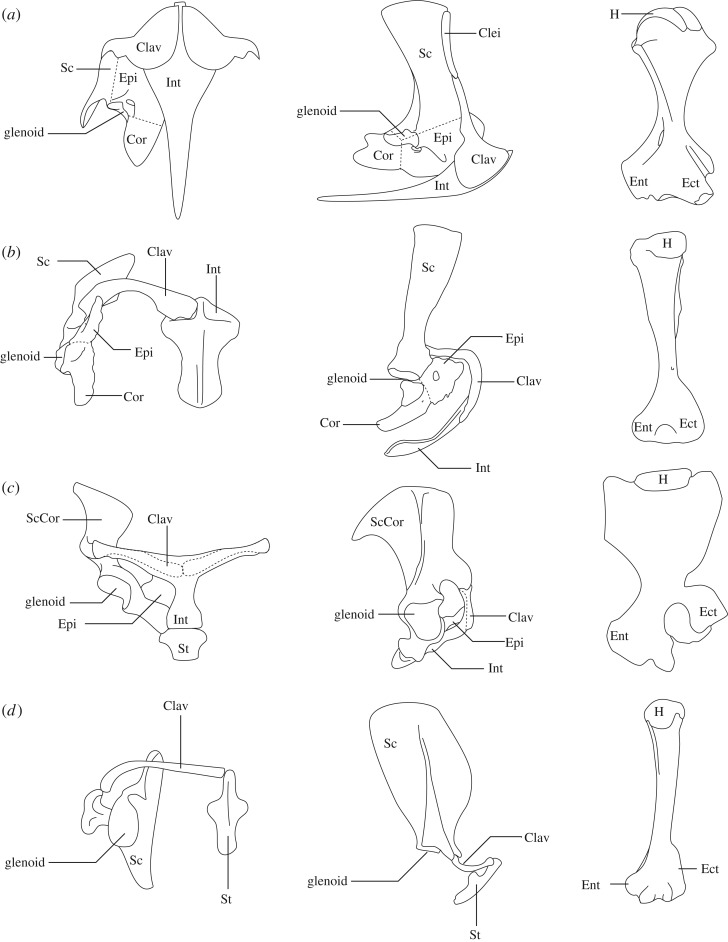
Schematic illustration of the pectoral girdle and humerus in different synapsid taxa (not to scale): (*a*) the ‘pelycosaur’ *Dimetrodon* right pectoral girdle in ventral and lateral view (modified from [4]) and right humerus in dorsal view (modified from [5]), (*b*) the cynodont *Massetognathus* right pectoral girdle in ventral and lateral view and humerus in dorsal view (modified from [6]), (*c*) the echidna *Tachyglossus* right pectoral girdle in ventral and lateral view (modified from [7]) and right humerus in dorsal view (modified from [8]), (*d*) the metatherian *Didelphis* right pectoral girdle in ventral and lateral view (modified from [7]) and humerus in caudal view. Note the cranial-most coracoid element is called ‘epicoracoid’ here (consistent with the terminology we have used for the echidna), but is sometimes referred to as ‘precoracoid’ or ‘procoracoid’ in other publications. Abbreviations: Clav, clavicle; Clei, cleithrum; Cor, coracoid; Ect, ectepicondyle; Ent, entepicondyle; Epi, epicoracoid (also known as procoracoid, precoracoid); H, humeral head; Int, interclavicle; Sc, scapula; ScCor, scapulocoracoid; St, sternal element.

The remarkable taxonomic and ecological diversity of therian mammals (marsupials and placentals) is underpinned by exaptation of the forelimb to serve novel locomotory styles and behaviours, e.g. cursorial horses, volant bats, aquatic whales, fossorial moles [1]. Such diversity of forelimb function was made possible due to a profound reorganization of the ancestral synapsid musculoskeletal system over the course of approximately 300 million years [2,3] (figure 1). The earliest synapsids (‘pelycosaurs’) had bulky and constrained U-shaped pectoral girdles, screw-shaped glenohumeral joints that limited humeral motion to a proscribed pathway, and short, horizontally oriented (‘sprawling’) limbs [2] (figure 1*a*). In contrast, therian mammals possess reduced and highly mobile pectoral girdles, ventrally oriented ball-and-socket glenohumeral joints, and upright (‘parasagittal’) limb posture and movement [7] (figure 1*d*). The transformation from the ancestral synapsid-style to the modern therian-style forelimb is dramatic, but the sequence of anatomical changes, their functional consequences, and the influence of forelimb reorganization on mammalian diversification are unclear [2,9].

The egg-laying monotreme mammals share a common ancestor with therian mammals, and so offer insight into an important stage of synapsid forelimb evolution. Although monotremes have been evolving independently of other crown mammals for over 166 million years [10], with some musculoskeletal features probably related to their specialized fossorial and aquatic lifestyles [7,9,11], they have many plesiomorphic traits in common with stem mammaliaforms (*sensu* [7]) and non-mammalian synapsids, e.g. sprawling-type gait, numerous large pectoral girdle bones (the coracoid, epicoracoid and interclavicle), immobile clavicle–interclavicle joint, a hemi-sellar (half saddle-shaped) laterally facing glenoid, and absence of the scapular supraspinous fossa [3,7,12–15] (figure 1*c*). The configuration of the pectoral girdle and glenohumeral joint in monotremes (and stem mammals) is thought to constrain mobility of the forelimb, but provide greater shoulder stability [2,7,9]. Reduction of the pectoral girdle throughout mammalian evolution and acquisition of a ball-and-socket shaped glenoid in therian mammals is inferred to have increased mobility, but consequently required more active muscular effort to stabilize the joints [2,7,16,17]. However, the increased shoulder mobility enabled functional versatility and allowed the forelimb to be brought beneath the body, which may have conferred several benefits to therians including increased efficiency [18] and/or agility [2].

Monotremes are crucial in building a complete picture of mammalian forelimb evolution, but the clade is depauperate—represented by only three extant genera (the platypus, and short-beaked and long-beaked echidnas) and a poor fossil record [19]. Due to their relatively limited distribution and the endangered status of some species, monotremes have been subject to only a handful of locomotory studies [14,20–22], leaving many aspects of their functional anatomy unexplored. Here we build a musculoskeletal computer model of the pectoral girdle and forelimb in the short-beaked echidna *Tachyglossus aculeatus* to estimate maximal joint ranges of motion and muscle moment arms—fundamental descriptors of musculoskeletal geometry and function [23]. We use outputs from the model to test and quantify inferences regarding pectoral girdle mobility and stability in echidnas, as well as muscle function, and compare these to extinct taxa with similar anatomy to gain insight into mammalian forelimb and locomotor evolution.

## 2. Material and methods

### 2.1. Acquisition of skeletal morphology

The musculoskeletal model was based on a 3.3 kg skeletally mature female short-beaked echidna, *Tachyglossus aculeatus*, obtained from the University of Adelaide. Cause of death was suspected impact with a vehicle, and the specimen showed a rostral fracture to the beak. Skeletal maturity was judged by fusion of long bone epiphyses through radiographic imaging (70 kW, 228 µA); no other obvious fractures were observed. The specimen was collected an unknown period of time after death and stored frozen at −18°C.

Skeletal morphology was acquired on the intact specimen using a HMXST225 micro-CT system (X-Tek, Amherst, NH, USA) at 160 kV, 165 µA, voxel size 0.127 mm, and with a 1 mm copper filter. The CT projections were reconstructed as a TIFF image stack using CT Pro 3D software (Nikon Metrology Inc., Brighton, MI, USA), imported into Mimics version 19.0 (Materialise, Leuven, Belgium), and the bones segmented into three-dimensional surface meshes to be exported as eight .stl files. The cervical and thoracic vertebrae, ribs and sternal elements were exported together as one mesh (the ‘axial’ segment), with the following bones exported as separate meshes: fused interclavicle–clavicle, left epicoracoid (also called the procoracoid), left scapulocoracoid (as the ‘scapula’ segment), the left humerus (the ‘upper limb’ segment), and left radius, ulna and manus (separate meshes but combined later into the ‘lower limb’ segment).

### 2.2. Measuring joint spacing

The specimen was kept intact to preserve joint spacing as altering joint distances can affect model-estimated ranges of motion and muscle moment arms (e.g. [6,23]). Joint spacing values are not recorded for many species (including monotremes, as far as we are aware), but are useful to record for future modelling of disarticulated specimens and estimation of appropriate spacing in extinct animals. Joint spaces were measured by importing three-dimensional bone models into Meshmixer (Autodesk, San Rafael, CA, USA), where the meshes were edited to remove everything but articular surfaces, including removal of the edges of the articular surfaces. These were then imported into Navisworks Manage 2018 (Autodesk, San Rafael, CA, USA) and the ‘measure shortest distance’ function was used to calculate the minimum distance between two bones' articular surfaces on either side the joint. This approach reduced the risk of accidentally selecting and measuring oblique distances between joint surfaces and/or overestimating cartilage thickness (if the articular surfaces are not in contact at the measured point).

Articular cartilage thickness at various joints in therian mammals has been found to correlate with body mass [24,25], raising the possibility of estimating joint spacing in disarticulated specimens when body mass (or a reliable proxy) is known. As a preliminary test of whether the body mass scaling relationships for cartilage thickness at the shoulder (glenohumeral) and elbow (humeroradial) joints [24] hold true for monotremes as well as therians, we infer articular cartilage thickness in echidna joints. The minimum joint distances measured above were halved, and these values were used in calculating whether the shoulder and elbow joints in our echidna fell within the 95% confidence interval of the regression based on therian mammals [24].

### 2.3. Joint axes, range of motion and anatomical reference pose

The bone segment meshes were imported into 3ds Max 2018 (Autodesk, San Rafael, CA, USA) in their original, articulated configuration (i.e. no translations were done so as to preserved joint spacing). The bones were assembled into a kinematic hierarchy, whereby subordinate distal segments inherit the translations and rotations of their proximal parent in the hierarchy. The manus, ulna and radius together occupy the most subordinate position in the hierarchy, and were parented to the humerus. The humerus was parented to the scapulocoracoid, as was the epicoracoid. The scapulocoracoid was parented to the clavicle–interclavicle and the axial segment, which together occupied the most superior position in the hierarchy.

**Figure 2.**
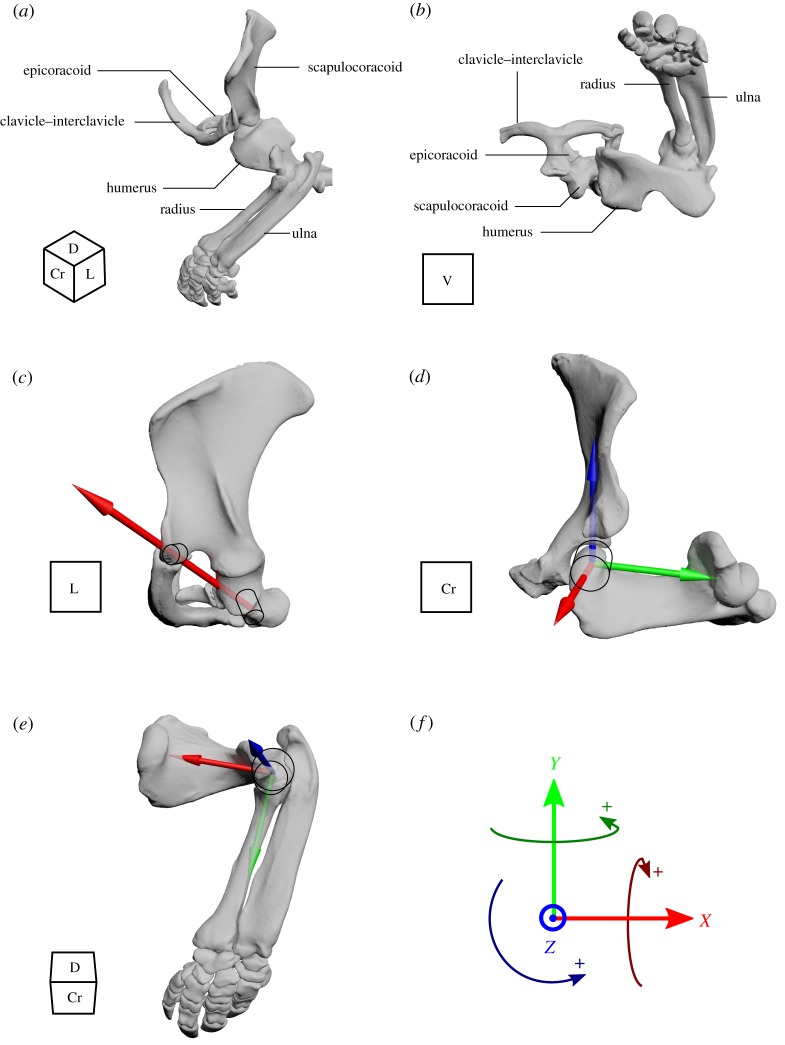
Left pectoral girdle and forelimb of the echidna *Tachyglossus aculeatus* in the anatomical reference pose: (*a*,*b*) Whole limb views of the reference pose. Joint axes and schematics of the primitive shapes used to define joint centre are shown for the (*c*) scapulocoracoid–clavicle–interclavicle joint modelled with 1 d.f. and two points of articulation, (*d*) glenohumeral joint modelled with 3 d.f., and the (*e*) humeroradioulnar joint modelled with 3 d.f. and a joint centre midway between humeroradial and humeroulnar primitives. Joint rotations follow the right hand rule, where the positive direction of rotation is counterclockwise when viewed from the top of the axis (*f*). For the 3 d.f. joints, rotation around *X* is defined as abduction–adduction, around *Y* as long-axis rotation, and around *Z* as flexion–extension. The cube in the bottom left of panels denotes anatomical orientation with reference to the whole animal: Cr, cranial; D, dorsal; L, lateral; V, ventral.

To assign joint coordinate systems (JCS), joint centres were established by fitting geometric shapes (‘primitives’) to the articular surfaces of the pectoral girdle and forelimb joints (i.e. the ‘geometric method’, e.g. [23,26]). The size, shape and position of the primitives were manipulated within 3ds Max until the primitive surface matched as closely as possible the bone contours of the joint surface, as judged by eye. Joints were then manipulated via their axes until bone-to-bone collision occurred to estimate range of motion (ROM), and positioned in an anatomical reference pose (figure 2*a*,*b*). The following section details the process for determining the JCS for each joint.

The fused clavicle–interclavicle is anchored to the body via the sternum, and articulates with the scapulocoracoid at two points: at the acromioclavicular joint and the interclavicle–coracoid joint [7,13]. Cylinder primitives were fitted to the articular surface of the acromion (for estimation of the acromioclavicular joint centre) and coracoid (for the joint centre between the interclavicle, coracoid and presternum/manubrium). Because the scapulocoracoid and fused clavicle–interclavicle are effectively two bones joined at two points, and assuming only rotation at the joints, in effect the scapulocoracoid is only permitted a 1 degree-of-freedom (d.f.) hinge-like rotation around an axis connecting the two joint centres. We call this joint the scapulocoracoid–clavicle–interclavicle (figure 2*c*).

The articular surface between the scapulocoracoid and the epicoracoid is essentially flat and roughly rectangular in shape. A rectangular plane primitive was fit to the articular surface of the epicoracoid, with the plane midpoint as the estimated joint centre. For the model, this joint was assumed to be a 1 d.f. hinge joint, allowing rotation of the epicoracoid around the long axis of the plane, so that the epicoracoid remained in contact with the interclavicle (to which it is loosely attached [7]). This was achieved by defining the scapulocoracoid–epicoracoid joint angle as a function of scapulocoracoid–clavicle–interclavicle joint angle.

For the glenohumeral joint, a cylinder primitive was fitted over the humeral articular surface and modelled as a 3 d.f. joint about three rotational axes (figure 2*d*). The joint axes were defined based on the bone and joint anatomy as follows: the proximodistal long axis of the humerus was defined as the axis connecting the glenohumeral and humeroulnar joint centres, and called the ‘*Y*’ axis (around which long-axis rotation occurs). A second axis was defined as orthogonal to ‘*Y*’ and parallel to the humeral cylinder primitive's long axis; it roughly corresponds to a craniocaudal axis, and is here called the ‘*X*’ axis (around which abduction–adduction occurs). The third axis was defined as orthogonal to ‘*X*’ and ‘*Y*’; it roughly corresponds to a dorsal–ventral axis and is here called the ‘*Z*’ axis (about which flexion–extension occurs). Range of motion was determined separately for each degree of freedom/axis (i.e. abduction–adduction, long-axis rotation and flexion–extension); see further below.

The elbow was set up as a 3 d.f. joint (figure 2*e*). Sphere primitives were fit to the radial and ulnar articular surfaces of the distal humeral common condyle to estimate the humeroulnar and humeroradial joint centres. Separate joint centres were used in the first instance to estimate separate flexion–extension ranges for each bone, because using a single common joint centre resulted in bone–bone contact at small changes in flexion–extension angle and thus (subjectively judged) implausibly low ranges of motion. We then combined the radius and ulna as one ‘lower limb’ segment, with the elbow joint centre defined as the midpoint between the radial and ulnar articular primitives. The proximodistal long axis of the lower limb segment was defined as the axis connecting the elbow and wrist joint centres (‘*Y*’ axis, about which long-axis rotation occurs). A second axis (‘*Z*’) was defined orthogonal to ‘*Y*’ and parallel to the elbow flexion–extension axis (as inferred from the anatomy of the common condyle and radial facet of the distal humerus). The third (‘*X*’) axis was defined as orthogonal to ‘*Y*’ and ‘*Z*’, passing through the short transverse axes of both the radius and ulna (about which abduction–adduction occurs).

Once all joints were assigned a JCS, each joint's ROM was determined through rotation around its joint axes, and an anatomical reference pose was constructed (figure 2*a*,*b*). Here we use a similar methodology to the ‘neutral pose’ method [6], in which the joints are placed at the midpoints of their estimated ranges of motion and set to 0°. The method for each joint is described in the paragraphs below.

The scapulocoracoid was rotated around its single axis with the clavicle–interclavicle until there was bony contact at either the acromioclavicular joint or the interclavicle–coracoid joint. The scapulocoracoid was then positioned in the middle of this range, defined as 0°.

For 3 d.f. joints like the shoulder (glenohumeral) and elbow (humeroradioulnar) joints, range of motion was determined by using the standard ‘independent rotations’ approach, which measures motions along each of the three orthogonal axes separately (e.g. abduction–adduction, long-axis rotation and flexion–extension). As one rotational axis is being measured the joint's other two rotational axes (and the other joints within the limb) are held fixed. The ‘midpoint’ could in theory depend on the axes' order of rotation and on the original scanned position of the joint. To try and account for these factors, iterative rotation and positioning to the midpoint of each ‘*X*’, ‘*Y*’ and ‘*Z*’ axis was undertaken until the adjustments were less than 1°. If the original position of the bone was altered beforehand via rotation about its joint axes, the process sometimes required a different number of iterations (e.g. five versus four for the glenohumeral joint if its axes were first aligned to world zero) but the final position was very close to the one if manipulated from the scanned position. For elbow flexion–extension ROM, we used the most conservative (restrictive) maximum and minimum values for the separately estimated radius and ulna. Adjustments around the elbow abduction–adduction and long axes were minimal, being already under 1° in the scanned position.

Recently, an automated workflow has been developed in Maya (Autodesk, San Rafael, CA, USA) to calculate maximal possible range of motion using simultaneous combined incremental rotations about each movement axis to generate thousands of poses, which are then checked for bone–bone contact and designated as ‘viable’ (no contact) or ‘non-viable’ (contact) [27]. We apply this ‘combined rotations’ approach to the glenohumeral joint to determine its impact on estimating maximal joint range of motion. To accomplish this, the echidna humerus was animated in Maya to sample combinations of glenohumeral rotations (−90° to 90° abduction–adduction, −180° to 180° long-axis rotation, −180° to 180° flexion–extension) at a resolution of 5°, yielding 197 173 poses.

### 2.4. Muscle geometry

**Figure 3.**
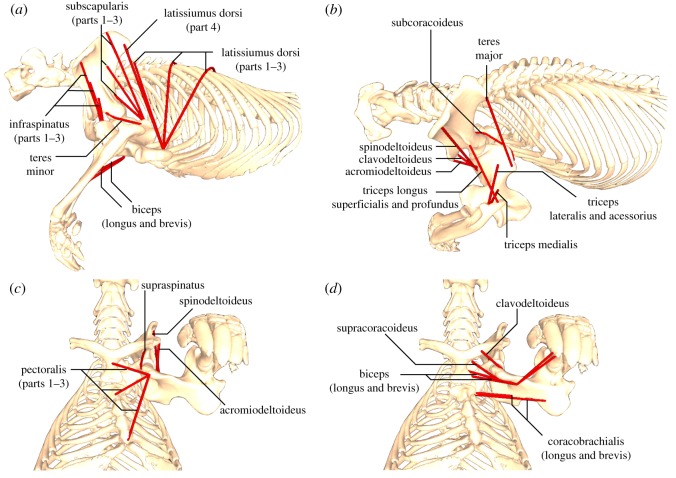
Musculoskeletal model of the echidna pectoral girdle and forelimb showing muscle geometry (origins, insertions, paths): (*a*) left lateral view, (*b*) left dorsolateral view, (*c*,*d*) ventral views. Note that specific muscles shown in each view have been chosen for clarity; these are minimally overlapping muscles, not superficial/deep layers. Some muscles are shown in more than one view.

Thirteen major muscles or muscle groups crossing the shoulder joint were included in the model (figure 3): *m. latissimus dorsi* (equivalent to *m. spinalis dorsi sensu* [8]), *m. pectoralis* (not divided as in therians; *sensu* [8]), *mm. deltoideus* (*sensu* [8], with three divisions: *m. clavodeltoideus*, *m. spinodeltoideus*, *m. acromiodeltoideus*), *m. subcoracoideus*, *m. supracoracoideus*, *m. infraspinatus*, *m. supraspinatus*, *m. teres minor*, *m. teres major*, *m. subscapularis*, *mm. coracobrachialis* (*sensu* [8], two divisions: longus and brevis), *mm. biceps* (two divisions: longus and brevis) and *m. triceps* (*sensu* [8], five divisions: longus profondus, longus superficialis, lateralis, accessorius and medialis).

The echidna specimen was kept intact for a future study and thus not available for dissection at the time of model-building. Instead, muscle origin and insertion data were taken from the comparative myological dissections of [8, figs 19B1-B2, 20B1-B2, 21B1-B4, 22B1-B4]. In 3ds Max, markers (‘point helpers’) were placed on the bone surfaces corresponding to the approximate centroid of the shaded attachment areas shown in these figures, judged by eye. The coordinates of the markers were used to define muscle lines of action during musculoskeletal model building, as described below. The divisions of large muscles (e.g. *m. coracobrachialis* longus and brevis) were modelled as separate lines of action. Muscles with broad attachment areas were also modelled as multiple, separate lines of action: *m. latissimus dorsi*, *m. pectoralis*, *m. subscapularis* and *m. infraspinatus* were modelled as three parts with separate lines of action originating from the approximate centroid and either extreme of the origin attachment area figured by [8]. These subdivisions were named parts 1, 2 and 3, respectively corresponding to the cranial-most, centroid, and caudal-most extent of the attachment site for muscles whose origins were broad in a craniocaudal direction (*m. latissimus dorsi* and *m. pectoralis*) or dorsal-most, centroid, and ventral-most extent of the attachment site for muscles whose origins were broad in a dorsoventral direction (*m. subscapularis* and *m. infraspinatus*). *M. latissimus dorsi* also has a fourth origin from the scapulocoracoid (named part 4). Finally, we combined the five divisions of *m. triceps* of [8] into three heads based on their similar geometry, modelled as three muscle lines (longus superficialis + profundus, lateralis + accessorius, and medialis).

To build the musculoskeletal model, bone segments, joint axes and muscle coordinates were imported into SIMM (Software for Interactive Musculoskeletal Modeling; [28]). Wrap objects were created to constrain the paths of the virtual muscles, preventing them from passing through the bone models during joint manipulation. As noted by previous authors [23], the decisions made when creating and assigning wrap objects are often subjective, and in some scenarios might result in the interpreted muscle function being significantly influenced by the user's wrapping choices. For this model, we aimed to create wrap objects that minimally altered the straight line paths of muscles, using what we judged to be the minimum wrapping required to prevent clipping of the muscles through bone surfaces. An exception was the wrap object for *mm. biceps*. The proximal humerus in *Tachyglossus* is wide and dorsoventrally flattened, and it was not immediately clear which side *mm. biceps* should naturally pass over (dorsal versus ventral). Here, a torus wrap object was used to constrain the proximal muscle path to the ventral side of the proximal humerus (as shown in [8, fig. 12B1]). All modelled muscle origins, insertions and paths are illustrated in figure 3 and an animation of the model is provided in electronic supplementary material, movie file S1.

### 2.5. Muscle moment arm analyses

SIMM's Plot Maker function was used to calculate individual muscle moment arms throughout the range of motion of each joint (as determined by the ‘individual rotations’ approach). Muscle actions and moment arms are described in terms of an unloaded limb. For example, *m. triceps* is described as an elbow extensor, though its role in locomotion is likely to be in resisting elbow flexion exerted by the ground reaction force. The individual muscle moment arm (MMA) data were then imported into Matlab (Mathworks, Natick, MA, USA) to determine the summed MMAs around the glenohumeral joint in abduction, adduction, internal rotation, external rotation, flexion and extension. Normalized summed MMAs were also calculated (as in [26]) by dividing these summed MMA values for each movement axis by the sum of each muscle's peak moment arm around that axis. As explored further in the Discussion, it is not clear which of these MMA metrics (individual MMAs, summed MMAs or summed normalized MMAs) are most useful—further testing of extant animal models with *in vivo* data is required—hence we have calculated and presented each metric in this study. To avoid muscles with multiple modelled origins or heads from being over-represented and artificially inflating the result, we calculated mean MMAs for *mm. biceps*, *mm. coracobrachialis*, *m. subscapularis*, *m. infraspinatus* and *m. latissimus dorsi* before all the MMAs were summed. The mean MMAs for the middle plus caudal parts (parts 2 and 3) of *m. pectoralis* were calculated, but the cranial part (part 1) was summed as a separate muscle. Similarly, *m. clavodeltoideus* was summed separately from the mean-averaged *m. spinodeltoideus* and *m. acromiodeltoideus*. This decision was based on electromyographic (EMG) muscle activation data from a monitor lizard that show the various parts of the *m. pectoralis* and *mm. deltoideus* have different activations and actions [17].

### 2.6. Sensitivity analyses

To examine the sensitivity of our musculoskeletal model to parametrization, we investigated whether changing certain input parameters altered the results and functional interpretations.

#### 2.6.1. Changing joint of interest reference pose

Usual practice for modelling 3 d.f. joints, like the glenohumeral joint, is to calculate MMA values as the joint rotates around one axis at a time (e.g. flexion–extension), while the other two axes (e.g. abduction–adduction, long-axis rotation) are fixed in the anatomical reference pose. However, altering the joint angles of the other two axes (e.g. altering the reference pose, so that the limb is maximally adducted or internally rotated) can alter the original calculated MMA values [29]. For practical purposes, there are too many joint angle and muscle combinations to test, but we examine the effect of changing the glenohumeral anatomical reference pose on the calculated MMAs of an important anti-gravity muscle, *m. pectoralis* part 2 (the line of action modelled through the middle of this muscle). Six alternative poses were explored—the reference pose was first altered so that the glenohumeral joint was maximally adducted, then abducted, then flexed, extended, internally rotated and externally rotated.

#### 2.6.2. Manipulating joints proximal/distal to joint of interest

Similar to above, usual practice in modelling studies is to calculate MMA values at the joint of interest, with the other joints fixed in their anatomical reference pose. However, the positions of the other joints also has the capacity to alter calculated MMA values (e.g. [26]), particularly for biarticular muscles. Here we examine the effect on the calculated moment arms of two important biarticular anti-gravity muscles crossing the glenohumeral joint (*m. pectoralis* and *m. triceps* pars longus) when the reference positions of the scapulocoracoid–clavicle–interclavicle joint and elbow joint are changed to be at either extreme of their ROMs. Four alternative poses were explored; two for each muscle. For *m. pectoralis*, the glenohumeral moment arms were re-calculated with the more proximal scapulocoracoid–clavicle–interclavicle joint at its most medial and most lateral positions. For *m. triceps* pars longus, the glenohumeral moment arms were re-calculated with the more distal elbow joint maximally flexed and maximally extended.

#### 2.6.3. Re-articulation of the digital skeleton

It is common practice to build musculoskeletal models from disarticulated museum specimens—meaning joint spacing is often unknown. In such instances, geometric primitives can be fit to both articular surfaces of a joint, and the centres of the primitives then aligned (bringing the bone meshes with them) to bring the bones into plausible articulation (e.g. *Massetognathus pascuali* glenohumeral joint [6]; *Tyrannosaurus rex* hip joint [26]). However, the primitive of one articular surface (e.g. the sphere fitted to the glenoid surface) can be larger than the other (e.g. the sphere fitted to the humeral head), and so bringing the two shapes and bones into alignment/articulation results in a offset (i.e. the difference in the radii of the two spheres) which may give an approximation of articular cartilage thickness [26]. Although our specimen was intact, we examine how digitally re-articulating the glenohumeral joint via alignment of primitives affects joint spacing, ROM and calculated MMAs for *m. pectoralis*.

## 3. Results

### 3.1. Joint spacing

The minimum joint distances varied between joints. The coracoid–interclavicle was most widely spaced (0.86 mm), then glenohumeral (0.7 mm), with acromioclavicle, humeroradial and humeroulnar joints having similar spacing (0.39, 0.36 and 0.32 mm). The coracoid–epicoracoid and epicoracoid–clavicle–interclavicle joints had very little to no space (0.13 and 0.00 mm respectively). The halved values (i.e. the inferred articular cartilage thicknesses) of the elbow joint followed the body mass scaling relationship for therian mammals, but the glenohumeral joint fell below two standard errors (electronic supplementary material, figure S1). At both joints, the inferred cartilage was thinner than would be expected from the scaling relationship established for equivalent joints in therian mammals [24].

### 3.2. Joint ranges of motion

Table 1 summarizes pectoral girdle and forelimb joint ROMs in each d.f., using the standard ‘individual rotations' approach. Modelling the two articulations between the scapulocoracoid and the clavicle–interclavicle (acromioclavicular joint and the coracoid–interclavicle joint) as a rotational hinge joint allowed a small amount of mediolateral scapulocoracoid movement before bone-to-bone contact, totalling 30°. At the glenohumeral joint, abduction–adduction had the greatest range of motion before bony impingement (total range 118°), followed by long-axis rotation of the humerus (54°), and flexion–extension (26°). At the humeroradioulnar joint, flexion–extension was greatest (total 114°), with long-axis rotation and abduction–adduction restricted to similar extents (22° and 20° respectively), though individually, the humeroradial joint showed slightly more flexion (5°) and the humeroulnar joint slightly more extension (−10°). These movements are also shown in electronic supplementary material, movie file S1.

**Table 1.** Ranges of motion about each joint axis of the echidna pectoral girdle and forelimb. The table shows the maximum possible rotations (in degrees) about each joint axis from the anatomical reference pose, before bone-to-bone contact.

	*X* (‘ab–adduction’)	*Y* (‘long-axis rotation’)	*Z* (‘flexion–extension’)
scapulocoracoid–clavicle–interclavicle	−15° to 15°	n.a.	n.a.
glenohumeral	−59° to 59°	−27° to 27°	−13° to 13°
glenohumeral (after re-articulation)	−31° to 57°	−20° to 9°	−10° to 10°
humeroradial	n.a.	n.a.	−57° to 62°
humeroulnar	n.a.	n.a.	−67° to 57°
humeroradioulnar	−10° to 10°	−10° to 12°	−57° to 57°

Of the 197 173 glenohumeral poses generated by the simultaneous ‘combined rotations’ approach [27], 6078 were found to be ‘viable’ (i.e. no bone contact). Compared with the ROMs estimated from the standard approach, this new method calculated similar ROMs but a larger envelope of movement (as shown in electronic supplementary material, figure S2). Notably, simultaneous rotations estimated greater humeral adduction; however, manual checking of the model showed that this adduction range was not anatomically feasible—to adduct the humerus beyond −59° as estimated using ‘independent rotations’, the humerus must be rotated approximately 180° around its long axis (i.e. sitting the wrong way up in the joint; electronic supplementary material, figure S2c). This means that ‘viable’ poses are not always anatomically realistic.

### 3.3. Muscle moment arms

**Figure 4.**
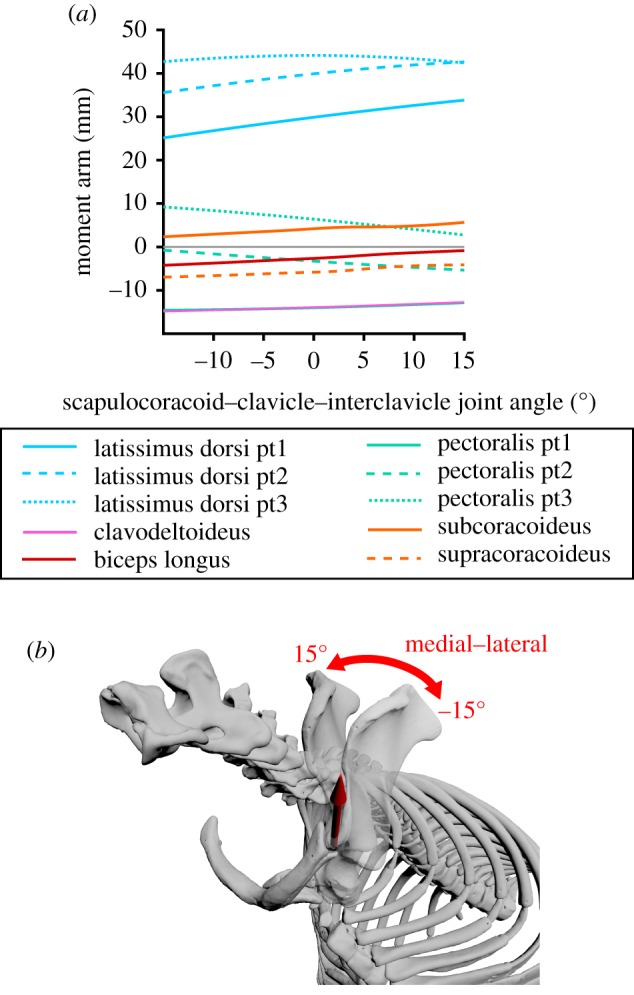
Model results for the scapulocoracoid–clavicle–interclavicle joint: Estimated moment arms from muscles crossing the scapulocoracoid–clavicle–interclavicle joint (*a*), throughout the scapulocoracoid–clavicle–interclavicle joint's range of motion (*b*).

**Figure 5.**
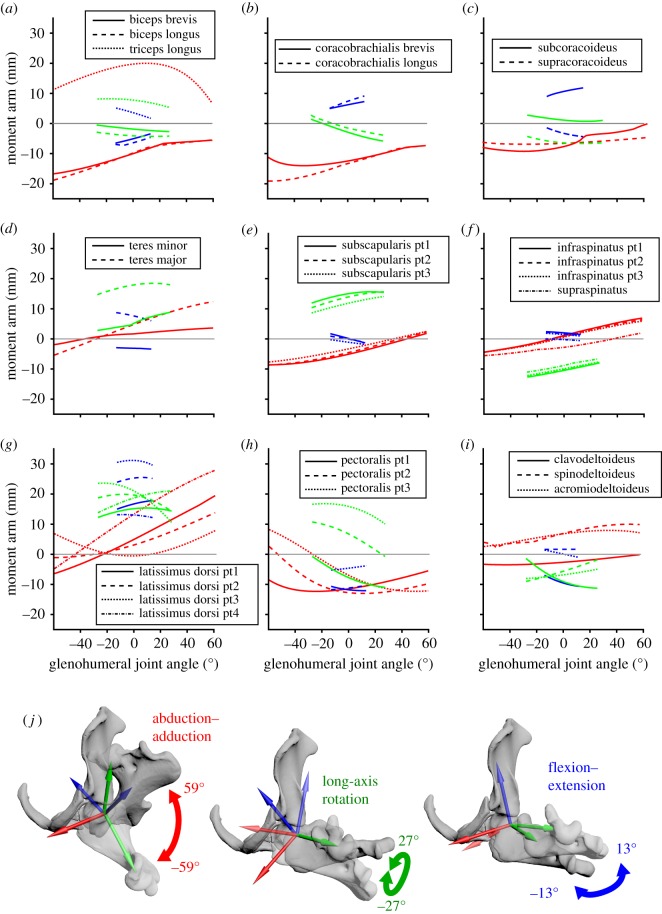
Model results for the glenohumeral joint: Estimated moment arms from muscles crossing the glenohumeral joint (*a–i*), throughout the glenohumeral joint's range of motion (*j*). Plotted colours correspond to rotation around each axis (red, abduction–adduction; green, long-axis rotation; blue, flexion–extension).

**Figure 6.**
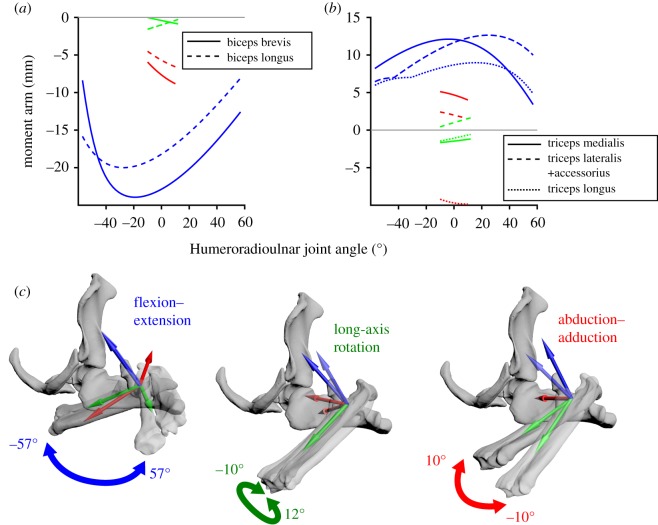
Model results for the humeroradioulnar joint: Estimated moment arms from muscles crossing the humeroradioulnar joint (*a*,*b*), throughout the humeroradioulnar joint's range of motion (*c*). Plotted colours correspond to rotation around each axis (red, abduction–adduction; green, long-axis rotation; blue, flexion–extension).

Plots of MMAs against joint angles for the modelled muscles are shown in figures 4–6. The relative magnitudes and sign (positive or negative) of MMAs indicate direction of rotation and therefore muscle action, summarized in electronic supplementary material, table S1.

#### 3.3.1. Muscle moment arms at the scapulocoracoid–clavicle–interclavicle joint

Six muscle groups are modelled crossing the scapulocoracoid–clavicle–interclavicle joint (figure 3): *m. latissimus dorsi*, *m. pectoralis*, *m. clavodeltoideus*, *mm. biceps* (longus), *m. subcoracoideus* and *m. supracoracoideus*. Most of these muscles are biarticular, additionally crossing the glenohumeral joint to insert on the humerus or radius/ulna. The MMAs around the scapulocoracoid–clavicle–interclavicle are shown in figure 4.

Parts 1–3 of *m. latissimus* (originating from the vertebrae) have large positive moment arms relative to other muscles crossing this joint, and indicate that *m. latissimus* acts to draw the scapulocoracoid medially. Part 4 of *m. latissimus* originates from the caudal edge of the scapulocoracoid, and only crosses the glenohumeral joint, thus its moment arm is zero.

The moment arms of *m. pectoralis* vary. Part 1, originating most cranially on the interclavicle shows large negative moment arms throughout the joint's ROM (relative to other muscles crossing this joint), indicating this region of *m. pectoralis* acts to produce stronger lateral scapulocoracoid movement. Part 2, originating from the presternum/manubrium, shows small negative moment arm values. Part 3, originating from the most caudal attachment site on the third sternal element, shows moderate positive moment arm values, indicating the converse action to part 1 (medial scapulocoracoid movement).

*M. subcoracoideus* shows small positive moment arms, indicating it acts to produce medial movement of the scapulocoracoid. *M. supracoracoideus* and *m. biceps* longus (both originating from the epicoracoid) show small negative moment arms and *m. clavodeltoideus* (originating from the clavicle) shows large negative moment arms, indicating respectively weak and stronger lateral scapulocoracoid movement produced by these muscle heads.

#### 3.3.2. Muscle moment arms at the shoulder (glenohumeral) joint

**Figure 7.**
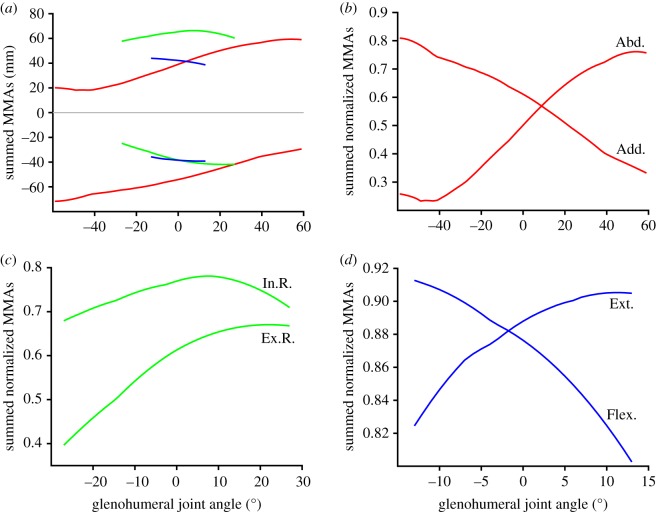
Summed muscle moment arms for all muscles crossing the glenohumeral joint: (*a*) summed muscle moment arms, (*b–d*) summed moment arms normalized by summed peak moment arms (i.e. summed moment arms/sum of maximum moment arm for each muscle). Plotted colours correspond to rotation around each axis (red, abduction–adduction; green, long-axis rotation; blue, flexion–extension). Abbreviations: MMAs, muscle moment arms; Abd., abduction; Add., adduction; In.R., internal rotation; Ex.R., external rotation; Ext., extension; Flex., flexion.

Thirteen muscles or muscle groups are modelled crossing the shoulder joint (figure 3): *mm. biceps* (longus and brevis), *mm. coracobrachialis* (longus and brevis), *m. subcoracoideus*, *m. supracoracoideus*, *m. triceps* (longus only), *m. teres major*, *m. teres minor*, *m. subscapularis*, *m. supraspinatus*, *m. infraspinatus*, *m. latissimus dorsi*, *m. pectoralis* and *mm. deltoideus* (*m. spinodeltoideus*, *m. clavodeltoideus*, *m. acromiodeltoideus*). The MMAs around the glenohumeral joint are shown in figure 5, and the summed moment arms at this joint are shown in figure 7.

Overall, abduction–adduction and long-axis rotation MMAs seem to predominate at the glenohumeral joint (figures 5 and 7). *Mm. biceps* and *m. coracobrachialis* show relatively large negative moment arms about the abduction–adduction (‘*X*’) axis and so are probably significant contributors to shoulder adduction, while *m. triceps* longus and *m. latissimus dorsi* show large abduction moment arms. *M. latissimus* also has large internal rotation and flexion moment arm values. *M. teres major*, *m. teres minor* and *m. subscapularis* have large internal rotation moment arms, while *m. infraspinatus* and *m. supraspinatus* have large external rotation moment arms.

Some muscles appear to have mixed actions. Moment arm values for *m. pectoralis* vary depending on the modelled origin, but taken together seem to show moderate shoulder adduction and extension, with some internal rotation. *M. supracoracoideus* has similarly sized adduction and external rotation moment arms, with smaller values for extension. *M. subcoracoideus* shows moderate flexion and adduction, with smaller internal rotation moment arms. The divisions of *mm. deltoideus* likewise have more mixed action; *m. spinodeltoideus* and *m. acromiodeltoideus* show moderate abduction and extension moment arms, and *m. clavodeltoideus* shows moderate extension and external rotation.

For muscles with broad origin attachment areas crossing the glenohumeral joint, the calculated MMAs changed between different origins, although the effect varied between muscles. For example, *m. infraspinatus* and *m. subscapularis* moment arm magnitudes only changed subtly depending on the modelled origin (figure 5*e*,*f*). Conversely, the role of *m. pectoralis* varied much more, with the moment arm magnitudes and even action changing between the cranial-most (part 1) and caudal-most (part 3) origins, e.g. switching from external rotation to internal rotation (figure 5*h*).

The summed moment arms for abduction, adduction and internal rotation have higher peak values than those of external rotation, flexion and extension (figure 7*a*). Both the summed moment arms (figure 7*a*) and the normalized summed moment arms (figure 7*b–d*) show similar patterns of peak moment arm versus joint angle. Shoulder abduction moment arm is maximized at maximally abducted joint angles, and the converse for adduction. The long-axis rotation angle that optimizes the internal and external rotation moment arms of the shoulder joint muscles are similar: at around 10° and 20° internally rotated from the anatomical reference pose, respectively. Flexor moment arms are greatest at maximal shoulder extension, and extensor moment arms are greatest at maximal flexion.

#### 3.3.3. Muscle moment arms at the elbow (humeroradioulnar) joint

Two muscles are modelled crossing the elbow (figure 3): *mm. biceps* and *mm. triceps*. Flexion–extension is the predominant action of these muscles, with *mm. biceps* exhibiting large negative moment arms (figure 6*a*) indicating flexion and *mm. triceps* large positive moment arms indicating extension (figure 6*b*). Both muscles also showed smaller moment arms around the ‘*X*’ axis (abduction–adduction) and very small long-axis rotation moment arms (figure 6).

### 3.4. Model sensitivity

#### 3.4.1. Changing joint of interest reference pose

Altering the anatomical reference pose of the glenohumeral joint differentially affected the calculated moment arms of *m. pectoralis* (part 2), depending on which axes were fixed and which were manipulated (electronic supplementary material, figure S3). Abduction–adduction moment arms varied in magnitude between maximally flexed and extended glenohumeral joint positions, and even more so between internally and externally rotated positions. However, the general pattern and inferred action (adduction) remained fairly consistent. This was not the case for long-axis rotation and flexion–extension moment arms, where changing the glenohumeral reference pose to be maximally abducted switched inferred action of *m. pectoralis* (part 2) from internal to external rotation and from extension to flexion.

#### 3.4.2. Changing reference pose at joints proximal/distal to joint of interest

Altering the scapulocoracoid–clavicle–interclavicle joint pose resulted in slightly different muscle moment arm magnitudes for *m. pectoralis* (parts 1–3) at the glenohumeral joint, but the pattern of moment arm versus joint angle and the interpreted actions remained the same (electronic supplementary material, figure S4). Altering elbow flexion–extension angle also slightly changed the muscle moment arms for *m. triceps* longus at the glenohumeral joint, such that glenohumeral flexion moment arm became larger than the internal rotation moment arm when the elbow was maximally flexed (although both still remained lower than the shoulder abduction moment arm; electronic supplementary material, figure S5).

#### 3.4.3. Re-articulation of the digital skeleton

Re-articulation of the glenohumeral joint via geometric primitive alignment resulted in a reduction of joint spacing distance compared with the original scanned position, from 0.7 to 0.23 mm. The effect of reduced joint spacing was to reduce the maximum ROM of the shoulder before bony contact at the articular surfaces (table 1; electronic supplementary material, figure S6). Adduction limits changed from −59° to −31° and abduction from 59° to 57°; external rotation from −27° to −20° and internal rotation from 27° to 9°; extension −13° to −10° and flexion from 13° to 10°. Re-articulation also slightly changed the magnitudes of calculated moment arms for *m. pectoralis* (electronic supplementary material, figure S6): re-articulation resulted in a peak moment arm difference of 18% for abduction–adduction, 11% for long-axis rotation, and 4% for flexion–extension. However, the patterns of moment arm versus joint angle remained almost identical and inferred actions were unchanged.

## 4. Discussion

Monotremes represent a key phylogenetic and anatomical stage in mammalian forelimb evolution, yet we still know relatively little about the biomechanical consequences of their unique morphology. To gain further insight into monotreme pectoral girdle and forelimb function, we created a musculoskeletal model of an echidna and used it to quantitatively investigate the influence of bony morphology on joint range of motion and muscle moment arms. The findings of our model are detailed below, with comparisons to available *in vivo* data from echidnas and therians. We conclude our discussion with anatomical comparisons to extinct taxa (from ‘pelycosaurs’ to stem therians), using our results and qualitative descriptions of fossil species to infer forelimb function during mammal evolution.

### 4.1. Joint spacing

Intra-joint spacing of the intact echidna varied between the joints of the forelimb, similar to other mammals that have been sampled [24]. The synovial joints (interclavicle–coracoid, glenohumeral, acromioclavicle, humeroradial and humeroulnar) had the greatest distance between bones, while the non-synovial joints (coracoid–epicoracoid and epicoracoid–clavicle–interclavicle) had little to no joint space, consistent with these joints having much less cartilage between the bones. Compared with available data from the shoulder (glenohumeral) and elbow (humeroradial) joints of therian mammals from [24], the echidna's inferred cartilage thickness was thinner than expected for its body weight—in the case of the glenohumeral joint, below the 95% confidence interval (electronic supplementary material, figure S1). When building musculoskeletal models of extinct mammals bracketed by monotremes and therians (and potentially other mammal relatives), spacing for some joints like the elbow could be estimated using the therian scaling relationship, provided body weight (or a proxy) is known. However, more data are needed for other joints (e.g. as has been done for the therian knee joint [25]) and other species (as noted by [30] and others), particularly outside of therian mammals.

Re-articulation of the glenohumeral joint via alignment of fitted primitives—as is sometimes done for extinct animals—reduced joint space in the echidna to 37% of the distance in the original scanned state. The reduction could be due to the difficulties in capturing full joint surface morphologies with simple geometric shapes. The reduced joint space also resulted in reductions in estimated ranges of motion (electronic supplementary material, figure S6), as has been observed in previous musculoskeletal modelling studies (e.g. [6,23]). This is also seen experimentally—for example, like the echidna, crocodylians have a sprawling-type gait and hemi-sellar glenoid [31], but have large epiphyses composed of articular cartilage [30] and much larger ranges of motion at the glenohumeral joint [32]. These results reinforce the need for care when accounting for the cartilage within the joints of musculoskeletal computer models, since intra-joint spacing has the capacity to greatly affect estimated maximal ROMs. The re-articulation method might be improved for extinct or disarticulated specimens by using primitive alignment to bring bones into approximate articulation, and then increasing the joint spacing with a scaling equation derived from body weight or another metric (as above).

### 4.2. Pectoral girdle and forelimb mobility

The echidna's scapulocoracoid is effectively fixed at two points to the clavicle–interclavicle and sternum; modelling these as a single 1 d.f. rotating joint allowed some (30°) mediolateral scapulocoracoid movement, as described qualitatively by [7]. The hemi-sellar shoulder (glenohumeral) joint in our model showed a large abduction–adduction range (118°) before encountering bony stops, a moderate amount of long-axis rotation (54°) and limited flexion–extension (26°). At the elbow (humeroradioulnar) joint, we estimate a large range of flexion–extension (114°), with small amounts of abduction–adduction and long-axis rotation (22° and 20°) (table 1). The ‘independent rotations’ method for estimating the range of motion (ROM) of 3 d.f. joints, an approach widely used in musculoskeletal modelling studies (e.g. [23]), appeared to capture much of the glenohumeral joint's mobility compared with the more complex recently developed ‘combined rotations’ workflow [27]. The more in-depth mapping of the latter is useful as a visual display of possible joint excursions, but requires careful implementation (restrictive input limits and/or manual checking) to eliminate ‘viable’ but anatomically impossible poses (electronic supplementary material, figure S2). With respect to model calculations of MMA, independent rotations about each joint axis are more suitable with current model building methodologies—among other things, modelling all combinations of joint rotations would require a prohibitive amount of muscle wrapping and manual checking of muscle paths.

Cineradiography has been used to estimate voluntary *in vivo* glenohumeral joint mobility in *Tachyglossus* [14,33]. Our results for glenohumeral long-axis rotation and flexion–extension are reasonably consistent with these estimations from experimental data (40–50° long-axis rotation and a ‘small amount’ of anteroposterior humeral movement, equivalent to our flexion–extension; [14]). Conversely, estimates of abduction–adduction at the glenohumeral joint do not closely match (15° [14] versus 118° here), and a combination of factors may be responsible. The *in vivo* kinematics were recorded in dorsal view only [14], which may have made abduction–adduction more difficult to estimate, particularly without correction of radiographic distortion, as is the norm in more recent studies (e.g. [34]). Voluntary locomotion behaviours, such as walking, are also unlikely to capture the maximum ROM available to the animal across its full behavioural repertoire (e.g. digging). We are also likely to be using subtly different limb axes to describe joint movement—ours are based on anatomical landmarks derived from detailed X-ray microtomography; the *in vivo* dataset also seems to use anatomical axes visualized through cineradiography [14], although these axes are not explicitly detailed. However, the most significant factor in the two divergent estimates is likely to be absence of soft tissues (e.g. ligaments, muscle volumes and skin) in our model. Previous studies have shown integumentary structures and muscles can greatly limit joint mobility (e.g. [32,35]) and the large difference between our model and *in vivo* estimations [14] of abduction–adduction, compared with other movements, suggest that the echidna's dorsal dermal spines and bulky subcutaneous and proximal limb musculature may significantly constrain limb movement.

A previous study [32] also estimated forelimb (shoulder, elbow) joint ROMs in a monotreme (the platypus *Ornithorhynchus anatinus*), using virtual bone models. The resulting estimated joint range values were different to our results, with large ranges for abduction–adduction, long-axis rotation and flexion–extension. However, there are several methodological differences between our studies, in line with the aims of [32]: in particular, that the models had a very permissive definition of possible poses, allowing for joint translation and variable joint spacing. To allow the models to be more directly compared, we re-positioned the platypus glenohumeral joint from [32] into a similar anatomical reference pose to the echidna in this study, and altered the joint spacing to match the echidna (fixed at 0.7 mm at the approximate mid-articular surface). The resulting ROMs using the approach here were in closer agreement with the results for the echidna: a relatively large range for abduction–adduction range (70°), slightly lower long-axis rotation (48°) and restricted flexion–extension (22°). As with the echidna, these ranges seem in reasonable agreement with experimental data from the platypus [20], with computer model abduction–adduction again exceeding *in vivo* voluntary ranges (estimated *in vivo* as 30–40° [20]) but matching estimates of long-axis rotation (40–60°) and flexion–extension (20–30°).

We find the echidna's skeletal anatomy appears to constrain some types of forelimb movement compared with therian mammals, but not necessarily others. The reduction in the size and number of shoulder girdle bones in therians has allowed increased mobility of the scapula relative to the body, whereas in echidna the scapulocoracoid is linked to the body via several articulations. The hemi-sellar glenohumeral joint seen in the echidna has also been hypothesized to constrain abduction–adduction and long-axis rotation compared with the ball-and-socket style joint of therians [7]. Here, however, our model shows that the bony morphology of the joint in echidna is not inherently restrictive to abduction–adduction or long-axis rotation—the former appears relatively unrestricted in our model, while our estimates of the latter match the *in vivo* long-axis rotation range measured using biplanar fluoroscopy in rats (used as representative of ancestral eutherians) [36]. In contrast, glenohumeral flexion–extension range was significantly limited by the echidna's bony anatomy when compared with both its abduction–adduction and long-axis rotation, and flexion–extension at the glenohumeral joint in the rat [36].

At the elbow, the echidna has a single humeral condyle for combined articulation of the radius and ulna plus a small radial facet [37,38] (figures 1 and 2). We find a comparable range of elbow flexion–extension to a representative therian (114° here versus 93° in the rat [36]). The echidna's elbow configuration is thought to permit long-axis rotation of the lower limb as a unit (with the displaced radius maintaining contact with the radial facet) while restricting independent rotations and abduction–adduction of the radius and ulna (since these actions would result in dislocation of one or both bones [37]). Our model does indeed permit a small amount of long-axis rotation, but also a similar amount of abduction–adduction. As with the glenohumeral joint, soft tissues may be partly responsible for the difference in descriptions of elbow joint mobility [37] e.g. the radial and ulnar collateral ligaments (fig. 8 in [37]) may restrict abduction–adduction while permitting a small amount of long-axis rotation.

### 4.3. Functional inferences from muscle moment arm values

The MMAs calculated here allow muscle actions in the echidna to be identified (electronic supplementary material, table S1), and also allow the inference of the main contributing muscles to joint motion (or resisting the opposite joint motion, as may be the case for stabilizing muscles). At the scapulocoracoid–clavicle–interclavicle joint (figure 4), *m. latissimus dorsi* (parts 1–3, originating from the vertebrae) is the main driver of scapulocoracoid movement in terms of moment arm, drawing the scapulocoracoid medially. It is counteracted by *m. clavodeltoideus* and *m. biceps* brevis, which draw the scapulocoracoid laterally, although these muscles have much smaller peak MMAs than *m. latissimus dorsi*. At the glenohumeral joint (figure 5), the largest abductor MMAs belong again to *m. latissimus dorsi* (particularly part 4, originating from the caudal scapulocoracoid) and *m. triceps* longus. The largest adductor MMAs belong to *mm. biceps* and *mm. coracobrachialis*. Although the *m. pectoralis* is considered a major humeral adductor in other sprawling animals (e.g. [39]), *m. pectoralis* does not have the largest peak glenohumeral adduction MMAs in the echidna, but this might be offset by its large volume and architectural properties. Many muscles contribute to internal rotation of the humerus: again *m. latissimus dorsi*, *m. teres major*, *m. pectoralis* (part 1, originating cranially) and *m. subscapularis*. The largest peak MMAs for external humeral rotation belong to *m. infraspinatus* and *m. supraspinatus.* Compared with MMAs for other movements, there seem to be fewer muscles with flexion–extension as a predominant action, and the moment arms for flexion–extension also seem to be generally lower. The largest flexor moment arms belong to *m. latissimus dorsi* and *m. subcoracoideus*, counteracted in extension by *m. pectoralis* (part 1) and *m. clavodeltoideus*. Only two muscles were modelled crossing the elbow (figure 6); as expected *mm. biceps* showed predominantly large flexor moment arms and *mm. triceps* large extensor moment arms. As mentioned previously, we must note that muscle actions are described here in terms of the unloaded limb, but when working against external loads, activation of a muscle may co-occur with other limb movements. For example, we describe *m. triceps* as an elbow extensor and interpret it to produce elbow extension when active in the unloaded limb, but it may also be active during elbow flexion in order to counterbalance ground reaction forces or other loads. Combined EMG and kinematic studies in the echidna would clarify the exact functions of muscles in different behaviours.

Previous myological studies have noted that forelimb muscle attachments and positions in the echidna can differ significantly from therian mammals [6,8,11]. Our model allows us to correlate some of the anatomical differences with functional consequences—and here, too, these functional shifts reflect the importance of long-axis rotation in echidna locomotion. For example, the therian *m. subscapularis* travels from the medial surface of the scapula to the medially facing lesser tubercle of the humerus; whereas in echidna it travels from the medial coracoid and epicoracoid and caudolateral surface of the scapula to the caudolaterally facing lesser tubercle of the humerus [8] (figure 3*a*). Correspondingly, the major action of *m. subscapularis* in therians is glenohumeral adduction (e.g. [40,41]); while in the echidna this muscle appears to be a major humeral internal rotator (see above). Similarly, in therians *m. supraspinatus* travels from the supraspinous fossa on the lateral scapula to the greater tubercle on the craniolateral aspect of the humerus; in echidna, it runs from the medial scapula to the greater tubercle on the craniolateral aspect of the humerus [8] (figure 3*c*). In therians the major action of *m. supraspinatus* is adduction and extension of the humerus (e.g. [40,41]), whereas in echidna this muscle is instead a major (in terms of moment arm) humeral external rotator. A final example: in therians, *m. teres minor* travels from the caudal (axillary) border of the scapula to the greater tubercle on the craniolateral aspect of the humerus; in echidna it travels from the cranial scapula, postaxially (caudal to the humerus) to insert on the lesser tubercle on the caudolateral aspect of the humerus [8] (figure 3*a*). The main action of *m. teres minor* in therians is flexion and external rotation of the humerus [41], whereas the MMA of this muscle in the echidna suggests the predominant actions are instead internal rotation and extension.

The predominance of abduction–adduction and long-axis rotation in the muscles crossing the glenohumeral joint is supported by the summed MMAs, which show greater internal rotation and adduction moment arms relative to other motions across much of the joint's ROM (figures 5 and 7). Our findings thus suggest that the configuration of the echidna's musculoskeletal anatomy has resulted in increased capacity for its muscles to produce humeral adduction and internal rotation at the glenohumeral joint. Optimization for humeral adduction and internal rotation makes sense in light of qualitative descriptions of the echidna's posture and gait from other studies. The echidna humerus is held laterally, parallel to the ground [14], and so must rely on glenohumeral adductors to resist gravity. The echidna's humerus has also been noted to undergo mostly long-axis rotation during locomotion [14], and humeral internal rotation contributes to the medial orientation of the manus (‘in-fingerness’; [21]). In particular, the latter seems to be an important component facilitating lateral support and side-to-side rolling of the trunk, compensating for a trunk that is too stout to use the lateral undulation characteristic of other sprawlers [21].

The importance of long-axis rotation at the shoulder joint may reflect constraints imposed by the echidna's evolutionary history and plesiomorphic aspects of its anatomy. The non-mammalian ‘pelycosaurs’, with similar and apparently even more restrictive pectoral and glenohumeral anatomy than the echidna (see discussion below), are similarly interpreted with long-axis rotation as the predominant movement of the humerus (e.g. [4,9,38]). However, significant long-axis rotation may also be related to the echidna's fossorial lifestyle and sprawling posture: the *Scalopus* mole, a fossorial therian, has a similarly abducted and internally rotated forelimb, and likewise has many muscles that act on the humerus to produce internal rotation, e.g. *m. latissimus*, *m. teres major*, *m. teres minor*, *m. infraspinatus*, *m. supraspinatus*, *m. subscapularis* [42,43].

It has been hypothesized that the configuration of the monotreme pectoral girdle and glenohumeral joint require little muscular stabilization, in contrast to the more mobile but unstable joints of therians [7]. Although we find the bony anatomy of the glenohumeral joint more permissive to abduction–adduction than previous descriptions (see above in Discussion), our model seems to support this mobility–stability trade-off. We find very few muscles in the echidna pectoral girdle and forelimb fit the pattern of intrinsic joint stabilizers (muscles with moment arms that, when plotted against joint angle, cross zero with a negative slope; [44]). Where an intrinsic stabilizer pattern is exhibited, only very small MMA values are involved: *m. pectoralis* part 3 (caudal part) exhibits intrinsic muscular stabilization in glenohumeral abduction–adduction, *m. acromiodeltoideus* and *m. subscapularis* for flexion–extension, and *m. coracobrachialis* for long-axis rotation (figure 5). We are not aware of equivalent MMA data for the therian forelimb to enable direct comparison of this stabilizing function between species, but therian hindlimb muscles have been shown to exhibit this property [44–46]. We would predict similar patterns in the therian forelimb, perhaps in line with muscle activity experiments that suggest many therian shoulder muscles act to stabilize the glenohumeral joint (e.g. [40]). A particularly interesting muscle to test would be *m. supraspinatus*, which is a small external humeral rotator in the echidna, but whose large size in therians is hypothesized to compensate for the reduced pectoral girdle and shallow glenoid [7], and has been inferred to counterbalance other shoulder muscles through abduction and extension [40].

MMA peak ranges have been evaluated by some authors as predictors of working joint range or limb posture, with unclear conclusions (e.g. [23,47]). Our results echo these studies i.e. that a relationship between MMA and limb posture or dynamic range is not straightforward [23]. For example, at the glenohumeral joint, we find moment arms around each axis peak at various joint angles depending on the muscle examined (figures 4 and 7); no consistent joint range appears to dominate, yielding no obvious clues about how the large estimated abduction–adduction range could be limited, nor suggesting a joint angle/posture that would be universally beneficial for all muscles. The summed MMAs also show peaks at different joint angles (figure 7). Both internal and external rotation peak around 10° of internal rotation, suggesting this is a favourable position for the echidna humerus to habitually adopt in terms of maximizing moment arms of the humeral rotators. In contrast, the moment arms of the summed adductors peak as the limb becomes more adducted (and vice versa for abduction), suggesting self-reinforcement for this movement, while summed extensor moment arms peak as the humerus flexes (and vice versa for flexors) suggesting negative feedback/self-stabilization of this movement. With more detailed *in vivo* kinematics, it may be possible to tease out a relationship between peak MMA joint ranges and habitual postures or locomotory function. Moving forward, it would be beneficial to include muscle physiological cross-sectional area in our musculoskeletal model (perhaps in weighting individual muscle contributions to the summed muscle moment [26]) to test whether muscle torques can provide further functional insights.

### 4.4. Sensitivity of model to parametrization

Our sensitivity analyses generally showed small differences to MMA with changing model parameters (electronic supplementary material, figures S3–S6), and suggest that calculated MMAs are most affected by changing the fixed position of a joint to an extreme of its mobility range. We found that altering the glenohumeral pose to be maximally abducted changed inferred actions of *m. pectoralis* during long-axis rotation and flexion–extension ranges, from an internal to an external rotator and from an extensor to a flexor (electronic supplementary material, figure S3). However, the inferred actions of *m. pectoralis* did not change with the other glenohumeral positions tested. In part, this may be due to the unrealistically broad estimated range of abduction–adduction, compared with the smaller (and more consistent with *in vivo* data) ranges for flexion–extension and long-axis rotation. Both joint and whole limb reference pose appear to have variable impact on calculated MMAs. Bearing these issues in mind when creating the model, we chose an anatomical reference pose in which limb joints were positioned at the middle of their estimated maximum range of motion; hopefully representing habitually adopted limb and joint positions and thus allowing us to characterize typical muscle function. Future work involving experimentally derived three-dimensional joint kinematics will allow these MMA estimations to be further refined, and may help in distinguishing model artefacts from real effects. For example, the moment arm magnitudes differed between the cranial-most (part 1) and caudal-most (part 3) origins of *m. pectoralis*, switching the main action of this muscle from an external to internal humeral rotator (figure 5*h*), but experimental data suggest that there are different functional muscle regions in *m. pectoralis* in the monitor lizard, with the caudal part acting as in the echidna to produce internal humeral rotation [17].

### 4.5. Insights into forelimb evolution and function in extinct taxa

It has long been debated how and to what extent monotremes can serve as appropriate living analogues to extinct stem mammals and non-mammalian synapsids [6,14,48]. The echidna has some anatomical features reminiscent of ‘pelycosaurs’ (a paraphyletic group of non-mammalian synapsids, of which the best known is probably the sail-backed *Dimetrodon*; see figure 1*a*). These features include the massive pectoral girdle (composed of clavicle and interclavicle) anchored to the thorax, the broad ventral plate formed in part by the procoracoid/epicoracoid, and similar humeral morphology (broad and short, held horizontally from the body, with greatly expanded epicondyles [49]). However, the ‘pelycosaur’ glenoid is screw-shaped, and appears to significantly constrain glenohumeral mobility, although there has been debate on the exact ROMs possible in *Dimetrodon* [9]. In general, it appears that humeral long-axis rotation was important for locomotion [4,9], paralleling the echidna, with the most recent estimate as 35.8° [4]. The other motions are likely to be even further constrained by the screw-shaped glenoid compared to the hemi-sellar glenoid in echidna. Abduction–adduction has been estimated as under 20° [5], although some studies propose a more adducted posture [50,51]. Flexion–extension is thought to be minimal [4], with estimates under 20° [5] or 50° combined with long-axis rotation [52]—the 26° range in the supposedly more mobile hemi-sellar joint of the echidna would suggest that the lower estimates in *Dimetrodon* are more probable. The elbow of pelycosaurs is unlike the echidna (or any other living tetrapod), being composed of two distinct articular facets, and interpreted to have a stabilizing function with very little movement [38].

Monotremes have more often been compared to non-mammalian therapsids (e.g. [11]), with caveats, as a basis for understanding therapsid locomotion, posture and biology. Shared features again include the proportionally larger sternal elements (compared to later therians) including extra elements (the interclavicle and epicoracoid), the hemi-sellar glenoid, an extensive infraspinous fossa, a small (or absent) supraspinous fossa and associated muscle, and similar humeral morphology [14]. Cynodonts, in particular, have invited many comparisons to monotremes (e.g. [14,53–55]), and have allowed many differences as well as similarities to be appreciated (see [6,9] and figure 1*b*,*c*). For example, cynodonts have less overlap of the clavicle and interclavicle, and no contact between the scapulocoracoid and sternal elements. The glenoid (though similar in morphology) is oriented caudolaterally rather than laterally [6,9]. The elbow is similar to the echidna, possessing an ulnar condyle on the distal humerus and radial notch for radio-ulnar articulation, but also possessing a separate condyle on the distal humerus for the radius with an incipient intercondylar groove [38]. In both echidna and cynodonts, the contact at the radial notch precludes independent rotation of the radius, and the ROMs at the elbow may have been similar [38].

An exciting aspect of musculoskeletal computer modelling is its potential for quantification and direct comparison of function resulting from these anatomical similarities and differences. A similar musculoskeletal model of the cynodont *Massetognathus pascuali* has been created to infer ROMs in the pectoral girdle and forelimb [6], allowing us to evaluate previously qualitative comparisons between these two taxa. Greater mobility was found at the scapulocoracoid–clavicle joint in *Massetognathus* [6] than we find in the echidna (3 d.f. including 40° of mediolateral movement versus 1 d.f. and 30° in the echidna), probably due to less contact and overlap of pectoral girdle bones. Glenohumeral joint mobility appears comparable, at least in terms of long-axis rotation (40° versus 54° in the echidna) and flexion–extension (30° versus 26°), though not abduction–adduction (40° versus 118°). Our model and that of *Massetognathus* [6] also reflect the difference in glenoid orientation, with the echidna's humerus positioned approximately perpendicular to the sagittal body plane in our anatomical reference (i.e. mid-range of motion) pose, versus caudally at a 45° angle in the equivalent neutral pose of *Massetognathus* [6]. Elbow flexion–extension range of motion does indeed appear comparable, with a combined conservative estimation of 125° in *Massetognathus* (versus 114° in echidna).

The actions and relative mechanical advantages of some muscles in representative ‘pelycosaurs’, cynodonts and ‘primitive’ (outgroup) tetrapods have been inferred from their attachment sites, and this series used to suggest evolutionary trends from synapsids to stem mammaliaforms [9,14]. Interestingly, in some aspects of muscle function, the echidna appears closer to ‘pelycosaurs’ than to cynodonts. *M. subcoracoscapularis* (homologous *to m. subscapularis* [56]) is inferred to produce internal rotation in both ‘pelycosaurs’ and cynodonts; here, we find the same action in the echidna (in contrast with therians, where it produces adduction [40,41]). However, cranial displacement of the scapular blade (and the origin of *m. subcoracoscapularis*) relative to the glenoid is proposed to have increased the velocity advantage of this muscle (i.e. allow greater rotation per unit of muscle contraction, but therefore decrease MMA) in cynodonts relative to the more basal ‘pelycosaurs’ [9]. Subjectively, the configuration of *m. subscapularis* in echidna appears closer to the arrangement in *Dimetrodon* than *Cynognathus* (fig. 43, p. 153 in [9]), with the muscle's line of action projecting caudally onto the broad lesser tubercle of the humerus. Therefore, we hypothesize that the relative moment arm of *m. subscapularis* in echidna is closer to that of a ‘pelycosaur’ than to a cynodont (or even therapsids more broadly).

It has also been suggested that *m. pectoralis* in non-mammalian synapsids plays an important role as an anti-gravity muscle, holding the body clear of the ground through powerful humeral adduction [9]. The deltopectoral crest (where *m. pectoralis* inserts) has been observed to be relatively larger in a representative cynodont (*Cynognathus*) than ‘pelycosaur’ (*Dimetrodon*), and therefore interpreted to produce increasingly more powerful humeral adduction during synapsid evolution [9]. The deltopectoral crest length in our echidna measured 34% of humeral length (as measured from the top of glenohumeral articular surface to bottom of ulnar condyle); close to the 36% of *Dimetrodon* and much less than the 55% of *Cynognathus*, suggesting that the adduction ability and/or function of *m. pectoralis* in the echidna is again closer to ‘pelycosaurs’. As well as plesiomorphic similarities, there are of course other morphological specializations related to the echidna's fossorial lifestyle: the strongly recurved dorsal scapular border suggests larger moment arms for *m. teres major*, commonly augmented in fossorial animals [14], and thus more powerful internal rotation of the humerus by this muscle compared with non-mammalian synapsids. Elucidation of MMAs in extinct taxa would help with more nuanced interpretations and comparisons of function, and in testing proposed evolutionary trends in muscle leverage (i.e. weaker internal humeral rotation by *m. subcoracoscapularis* and stronger adduction by *m. pectoralis*; [9]).

Many extinct mammaliaforms (e.g. *Sinoconodon*, *Haldanodon*) also show some similar anatomical features to the echidna (e.g. [7,55,57]) and are thus likely to share some aspects of pectoral girdle and forelimb function. However, the expansion of the pectoral girdle in the echidna (potentially linked to its fossorial lifestyle [7]) means mobility is probably more limited in the echidna compared to mammaliaforms. The coracoid process in *Haldanodon* and *Sinoconodon* is short and probably did not contact the sternal elements as in the echidna [7], and so these mammaliaforms would have had greater ROM at the scapulocoracoid–clavicle–interclavicle joint, beyond the single degree of freedom imposed by the two-articulation configuration in the echidna. The humeri of *Haldanodon* and *Tachyglossus* are strikingly similar, and *Haldanodon* is postulated to have had a monotreme-like sprawling gait [57]. However, the broader entepicondyle in echidna [57] suggests proportionally larger moment arms for *m. latissimus dorsi* (which produces mostly flexion and internal rotation in the echidna)*—*therefore, although potentially similar, *Haldanodon* may have used a less internally rotated/‘in-fingered’ gait.

Stem therians (theriiforms) also have similar features to monotremes [55,58]. For example, like the echidna and also the stem mammaliaforms mentioned above, *Fruitafossor* shares a hemi-sellar shaped glenoid and similar scapular morphology, with ROM at this joint inferred to be similar to monotremes [58]. However, the coracoid is smaller and *Fruitafossor* had no epicoracoid, so it was likely to have had greater mobility of the scapula relative to the body when compared with the echidna and earlier mammaliaforms [7]. Like the echidna, *Fruitafossor* had an elongated and medially pointed olecranon process; however, the relative size of the olecranon dwarfs even the echidna's [58], suggesting *Fruitafossor* had much larger moment arms for *mm. triceps* to extend the elbow.

Evolutionary trends in the leverage of shoulder muscles have been touched on [9], and similar (perhaps related) patterns in the partitioning of scapular and glenohumeral mobility across mammalian evolution are suggested by available data from this and other studies. Namely, humeral long-axis rotation appears to be important in the sprawling echidna (like ancestral ‘pelycosaurs’), but humeral flexion–extension and pectoral girdle mobility are highly constrained, and the shoulder muscles are optimized to produce strong adduction and internal rotation (this study). Cynodonts, potentially encompassing both sprawling and more upright postures, exhibit relatively similar mobilities around each glenohumeral axis [6], with apparently concomitant reduction in the leverage of ancestrally important humeral rotators (e.g. *m. subcoracoscapularis* [9]). Parasagittal therians exhibit essentially the opposite pattern of shoulder mobility to the echidna and stem mammals, with glenohumeral flexion–extension being the greatest motion. These preliminary observations suggest that throughout synapsid and mammalian evolution, accruement of anatomical and functional changes have increased pectoral girdle mobility, and increased the flexion–extension component of both glenohumeral mobility and leverage (i.e. increased humeral flexor/extensor MMAs) relative to abduction–adduction and long-axis rotation, to facilitate the acquisition of upright posture in crown therians.

## 5. Conclusion

By building a musculoskeletal model of an echidna pectoral girdle and forelimb, we have been able to quantify three-dimensional joint mobility and muscle function for the first time. In terms of joint mobility, our model demonstrates that the scapulocoracoid–clavicle–interclavicle joint has a constrained range of motion; the hemi-sellar glenohumeral joint permits extensive abduction–adduction, a moderate amount of long-axis rotation, and limited flexion–extension; and the humeroradioulnar joint allows a large degree of flexion–extension and is more limited in the other axes of movement. Muscle moment arm results indicate that the echidna shoulder is optimized for humeral adduction and internal rotation. Further, the predicted functions of many shoulder muscles in echidna differ from therians as a consequence of their geometry: more muscles in echidna have roles in humeral long-axis rotation. These patterns are consistent with the limited *in vivo* experimental data currently available for monotremes. As monotremes exhibit many mixed plesiomorphic features (e.g. interclavicle, epicoracoid, hemi-sellar glenoid) our data provide a foundation to begin to interpret the functional anatomy of extinct taxa, from non-mammalian synapsids to stem therians—a critical step in reconstructing the evolution of mammalian locomotor diversity.

## Supplementary Material

Supplementary table and figures

## Supplementary Material

moviefileS1.avi

## Supplementary Material

Model MMA data

## Data accessibility

The muscle moment arm datasets generated in this study are available as electronic supplementary material online, accompanying the article.
