# Supplementary material for: Pectoral girdle and forelimb musculoskeletal function in the echidna (Tachyglossus aculeatus): insights into mammalian locomotor evolution
Source: R Soc Open Sci. 2018 Nov 14;5(11):181400. doi: 10.1098/rsos.181400 (PMC6281926; doi:10.1098/rsos.181400)
Supplement: Supplementary table and figures [file rsos181400supp1.pdf]

Pectoral girdle and forelimb musculoskeletal function in the echidna (*Tachyglossus aculeatus*): insights into mammalian locomotor evolution

Authors: Sophie Regnault and Stephanie E. Pierce

Affiliation: Museum of Comparative Zoology and Department of Organismic and Evolutionary Biology, Harvard University, 26 Oxford Street, Cambridge MA, USA

sophie_regnault@fas.harvard.edu (corresponding author)

spierce@oeb.harvard.edu

Document includes:

- **Table S1:** Major actions of muscles in the echidna pectoral girdle and forelimb.
- **Figure S1:** Log-scaled articular cartilage thickness vs. log-scaled body mass at the shoulder and elbow joints.
- **Figure S2:** Glenohumeral range of motion determined using the 'combined rotations' method of [24].
- **Figure S3:** Sensitivity of *m. pectoralis* (part 2) glenohumeral moment arm estimates to glenohumeral joint angles.
- **Figure S4:** Sensitivity of *m. pectoralis* glenohumeral moment arm estimates to alterations in scapulocoracoid-clavicle-interclavicle joint angle
- **Figure S5:** Sensitivity of *m. triceps longus* glenohumeral moment arm estimates to alterations in humeroradioulnar joint angle.
- **Figure S6:** Sensitivity of m. pectoralis glenohumeral moment arm estimates to alteration in joint spacing following rearticulation.

Supplementary Table S1: Major actions of muscles in the echidna pectoral girdle and forelimb.

Muscle	Action
Latissimus dorsi	Strongly draw the scapulocoracoid medially<br><br>Strong glenohumeral flexion and internal rotation, moderate to strong glenohumeral abduction
Pectoralis (cranial, part 1)	Strongly draw the scapulocoracoid laterally<br><br>Moderate glenohumeral adduction and extension, with some glenohumeral external rotation
Pectoralis (middle and caudal, parts 2 and 3)	Weakly draw the scapulocoracoid laterally (part 2) or moderately medially (part 3).<br><br>Strong glenohumeral internal rotation, moderate glenohumeral adduction (some weak abduction), moderate glenohumeral extension.
Biceps	Weakly draw the scapulocoracoid laterally<br><br>Strong glenohumeral adduction, lesser amounts of glenohumeral extension and external rotation.<br><br>Strong humeroradioulnar flexion, moderate humeroradioulnar abduction, weak humeroradioulnar external rotation.
Subcoracoideus	Moderately draw the scapulocoracoid medially<br><br>Strong glenohumeral flexion, moderate glenohumeral adduction, weak glenohumeral internal rotation.
Supracoracoideus	Moderately draw the scapulocoracoid laterally.<br><br>Moderate glenohumeral adduction, weak glenohumeral external rotation and extension.
Clavodeltoideus	Strongly draw the scapulocoracoid laterally<br><br>Moderate glenohumeral extension and external rotation, weak glenohumeral adduction.
Spinodeltoideus	Moderate glenohumeral abduction and external rotation, weak glenohumeral flexion.
Acrodeltoideus	Moderate glenohumeral abduction and extension.
Coracobrachialis	Strong glenohumeral adduction, moderate glenohumeral flexion, weak glenohumeral external rotation.
Triceps	Strong glenohumeral abduction, moderate glenohumeral internal rotation, weak glenohumeral flexion.<br><br>Strong humeroradioulnar extension, moderate humeroradioulnar abduction (longus) or weak humeroradioulnar adduction (other heads), weak humeroradioulnar internal rotation (combined heads of lateralis and accessorius) or external rotation (other heads).

Teres major	Strong glenohumeral internal rotation, moderate glenohumeral flexion and abduction (some weak adduction).
Teres minor	Moderate glenohumeral internal rotation, weak glenohumeral extension and abduction.
Subscapularis	Strong glenohumeral internal rotation, moderate glenohumeral adduction.
Infraspinatus	Strong glenohumeral external rotation, weak glenohumeral flexion, mixture of weak glenohumeral adduction and abduction.
Supraspinatus	Strong glenohumeral external rotation, moderate glenohumeral adduction.

Muscle actions were inferred from muscle moment arm magnitudes and signs, and are described in terms of the unloaded limb. Qualitative descriptors (strong, moderate, weak) denote relative magnitudes of muscle moment arms across each joint.

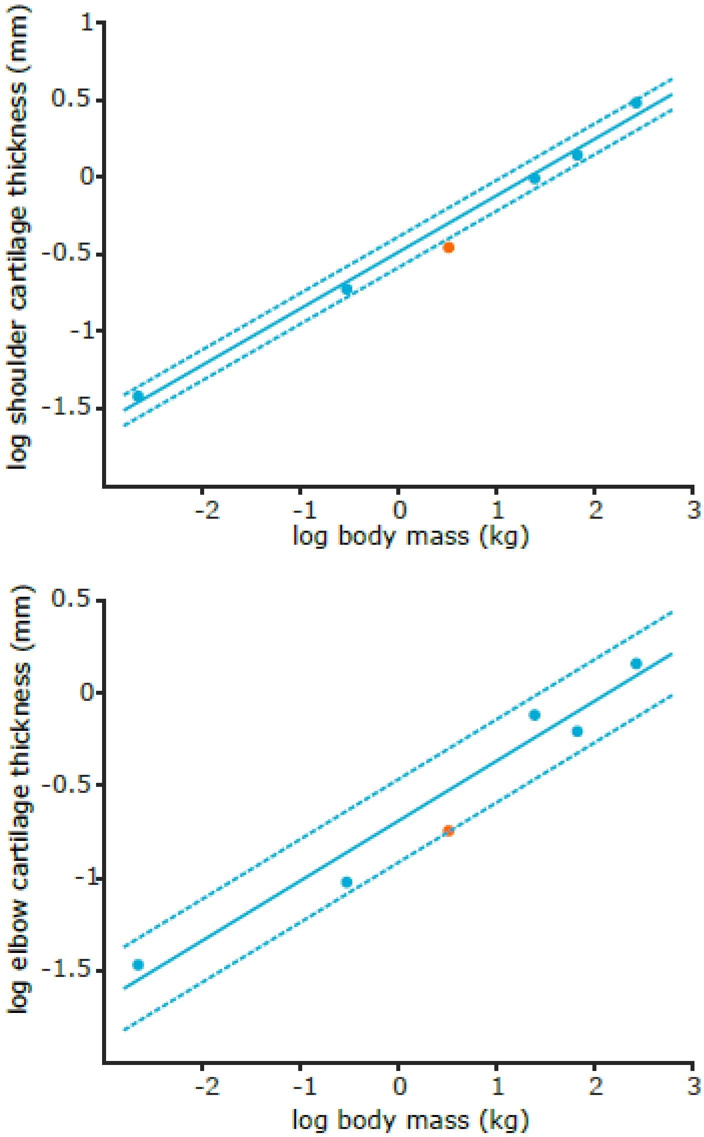

Figure S1. Log-scaled articular cartilage thickness vs. log-scaled body mass at the shoulder and elbow joints. The blue points denote the five eutherian species sampled by [21] (mouse, rat, dog, sheep, cow), with a line of best fit (solid line) and 95% confidence intervals (+/- 2 standard errors, dotted lines). The orange point represents measurements from the echidna (this study).

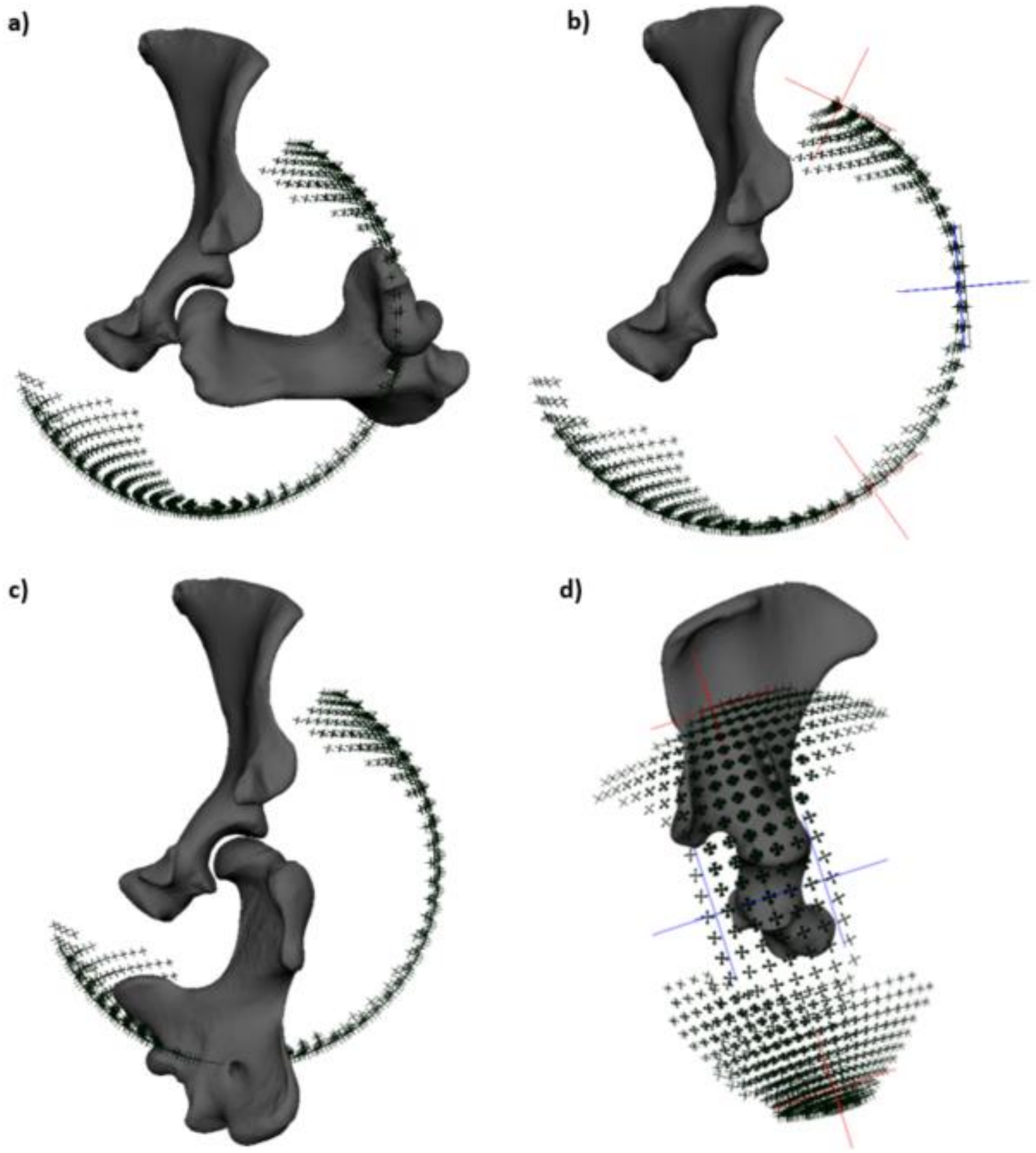

Figure S2. Glenohumeral range of motion determined using the 'combined rotations' method of [24]: a) Cranial view of glenohumeral joint in anatomical reference pose, with small black axes showing 'viable' positions of the distal humerus; b) Cranial view comparing results to the maximum range of movement determined by the 'independent rotations' method with red markers denoting the limits of abduction-adduction; c) Cranial view showing more adducted 'viable' positions are only possible with inversion of the humerus about its long-axis (i.e. it has been rotation ~180° from the anatomical reference pose); d) Lateral view comparing results to the maximum range of movement determined by the 'independent rotations' method with blue markers denoting limits of flexion-extension.

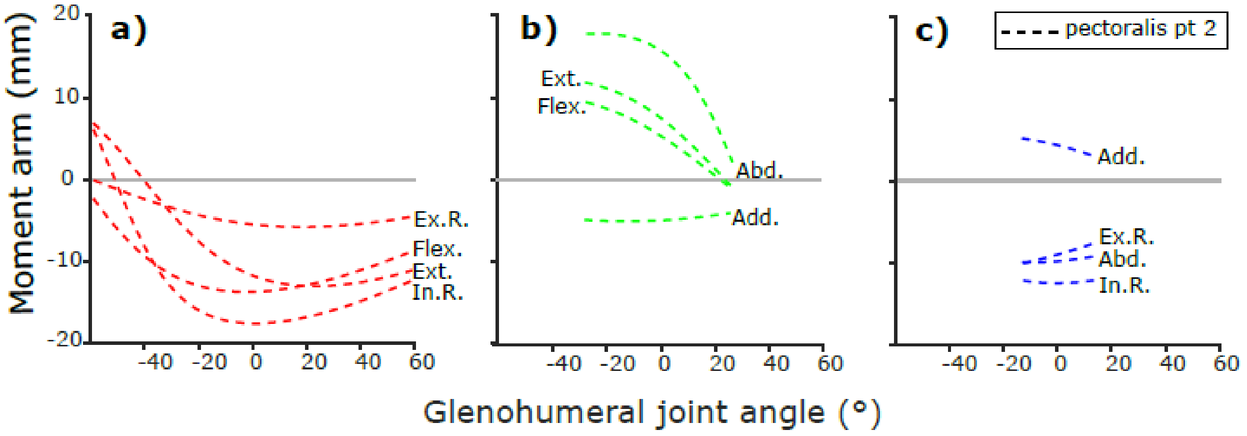

Figure S3. Sensitivity of *m. pectoralis* (part 2) glenohumeral moment arm estimates to glenohumeral joint angles: a) Abduction-adduction moment arms when the other two axes of the glenohumeral joint (i.e. flexion-extension and long-axis rotation) are fixed at either extreme of their range of motion; b) Long-axis rotation moment arms when the other two axes of the glenohumeral joint (i.e. flexion-extension and abduction-adduction) are fixed at either extreme of their range of motion; c) Flexion-extension moment arms when the other two axes of the glenohumeral joint (i.e. long-axis rotation and abduction-adduction) are fixed at either extreme of their range of motion. Ex.R. = maximum external rotation; In.R. = maximum internal rotation; Flex. = maximum flexion; Ext. = maximum extension; Abd. = maximum abduction; Add. = maximum adduction.

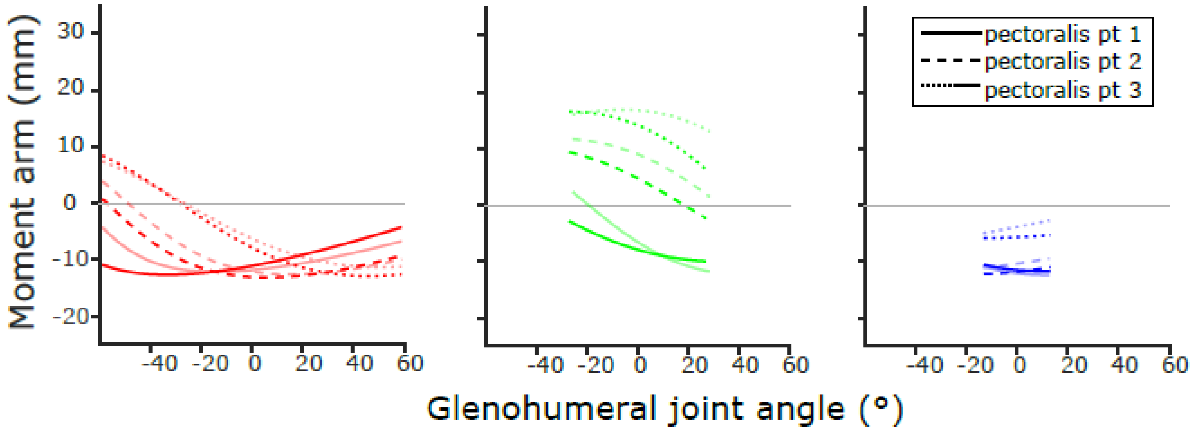

Figure S4. Sensitivity of *m. pectoralis* glenohumeral moment arm estimates to alterations in scapulocoracoid-clavicle-interclavicle joint angle: When the scapulocoracoid-clavicle-interclavicle joint is fixed at the medial extreme of its range of motion (ROM), muscle moment arm estimates follow the opaque plotted lines throughout glenohumeral ROM, versus the faded plotted lines when the scapulocoracoid-clavicle-interclavicle joint is fixed at the lateral extreme of its ROM.

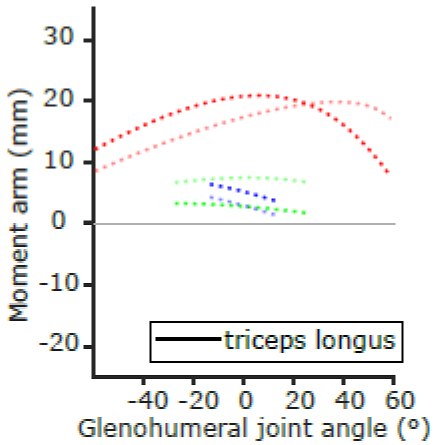

Figure S5. Sensitivity of *m. triceps longus* glenohumeral moment arm estimates to alterations in humeroradioulnar joint angle: When the humeroradioulnar joint is maximally flexed, muscle moment arm estimates follow the opaque plotted lines throughout glenohumeral ROM, versus the faded plotted lines when the humeroradioulnar joint is maximally extended.

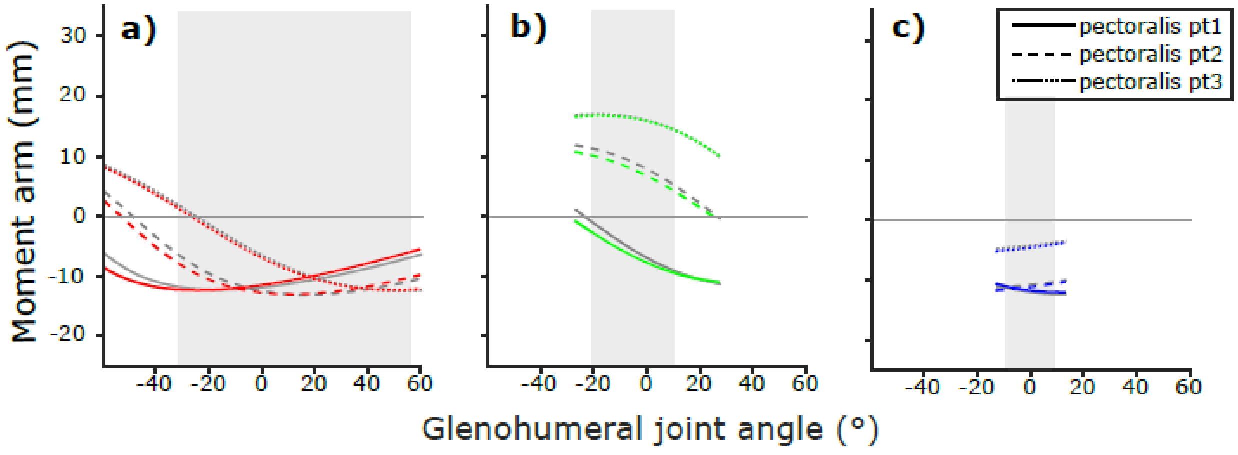

Figure S6. Sensitivity of m. pectoralis glenohumeral moment arm estimates to alteration in joint spacing following rearticulation: a) Abduction-adduction; b) Long-axis rotation; c) Flexion-extension. The primitive alignment method of joint rearticulation resulted in reduced ranges of motion for each joint axis (shown by gray bars), and slightly altered estimated muscle moment arms (shown by gray line plots), compared with the ranges of motion and moment arms in the original model (colored line plots).
